# A high-performance seizure detection algorithm based on Discrete Wavelet Transform (DWT) and EEG

**DOI:** 10.1371/journal.pone.0173138

**Published:** 2017-03-09

**Authors:** Duo Chen, Suiren Wan, Jing Xiang, Forrest Sheng Bao

**Affiliations:** 1 State Key Laboratory of Bioelectronics, Laboratory for Medical Electronics, School of Biological Science & Medical Engineering, Southeast University, Nanjing, China; 2 Division of Neurology, Cincinnati Children’s Hospital, Cincinnati, OH, United States of America; 3 Department of Electrical & Computer Engineering, University of Akron, Akron, OH, United States of America; Seoul National University College of Medicine, REPUBLIC OF KOREA

## Abstract

In the past decade, Discrete Wavelet Transform (DWT), a powerful time-frequency tool, has been widely used in computer-aided signal analysis of epileptic electroencephalography (EEG), such as the detection of seizures. One of the important hurdles in the applications of DWT is the settings of DWT, which are chosen empirically or arbitrarily in previous works. The objective of this study aimed to develop a framework for automatically searching the optimal DWT settings to improve accuracy and to reduce computational cost of seizure detection. To address this, we developed a method to decompose EEG data into 7 commonly used wavelet families, to the maximum theoretical level of each mother wavelet. Wavelets and decomposition levels providing the highest accuracy in each wavelet family were then searched in an exhaustive selection of frequency bands, which showed optimal accuracy and low computational cost. The selection of frequency bands and features removed approximately 40% of redundancies. The developed algorithm achieved promising performance on two well-tested EEG datasets (accuracy >90% for both datasets). The experimental results of the developed method have demonstrated that the settings of DWT affect its performance on seizure detection substantially. Compared with existing seizure detection methods based on wavelet, the new approach is more accurate and transferable among datasets.

## Introduction

Approximately 50 million people worldwide have epilepsy, making it one of the most common neurological diseases globally [1]. Epilepsy is characterized by recurring seizures caused by abnormal discharges in the brain [2]. Electroencephalogram (EEG), a technology directly records electrical activities from the brain, is an important data resource in epilepsy diagnostic tasks, such as, seizure detection [3, 4], spike detection [5, 6] and localization of epileptic foci [7, 8]. In clinical practice, long-term EEG recording up to a few days, is usually required. Therefore, many computer-aided solutions have been developed to assist neurologists. Combining signal processing and machine learning, most of those approaches model the problem as classification of signals, such as epileptic vs. healthy for epilepsy diagnosis [9, 10], ictal (on seizure) vs. inter-ictal for seizure onset detection [11, 12], etc. The most common classification problem is seizure detection, where seizure and non-seizure EEG segments of patients need to be identified [6].

Applying Discrete Wavelet Transform (DWT) on epilepsy-related EEG signal classification is gaining ground in recent years. The main advantage of DWT is that the resolution of time and frequency in DWT can be adapted to the frequency content of the examined patterns, thus leading to an optimal time-frequency resolution across all frequency ranges [13, 14]. This superiority makes DWT especially suitable for the analysis of non-stationary signal, such as EEG [6, 15].

Though DWT has shown promising results on seizure detection [6, 11, 16], it is still an open question regarding how to utilize the full potential of DWT to improve the accuracy and reliability of EEG analysis. Meanwhile, some methods only show promising results for selected patients, the reliability and reproducibility of the results have been questioned when being tested on other EEG datasets [17].

To establish a high-performance seizure detection algorithm based on DWT, the present study proposed a generalized computer-aided EEG analysis method to achieve the optimal seizure detection accuracy with low computational cost. Our method automatically searched the optimal combination of four factors, including, mother wavelet, decomposition level, frequency band, and DWT coefficient feature. These factors may affect the performance of DWT in seizure detection.

To test the performance of our method, we used EEG dataset from CHB-MIT (MIT) and dataset from University of Bonn (UBonn). Empirical results show that: 1) mother wavelet does not influence seizure detection results significantly; 2) seizure detection accuracy is very sensitive to decomposition level if the features of seizure/non-seizure EEGs showing significant difference in several frequency bands; 3) many frequency bands and DWT coefficient features are redundant causing accuracy reduction and unnecessary high computational cost.

Our seizure detection method achieved the accuracies of 92.30% and 99.33%, on MIT dataset and UBonn dataset, respectively. Compared with other seizure detection methods based on DWT, our approach attained the highest accuracy and the best robustness. The main innovation and contribution of the present study is the establishment of a guideline for constructing a high-performance seizure detection algorithm with high accuracy and low computational cost based on DWT and EEG.

## Method

### EEG datasets

We formulate the problem of seizure detection as classifying multi-channel EEG recordings (seizure and non-seizure). Some previous methods have shown promising results for selected patients; however, they achieved poor performance on other EEG datasets [17]. Considering this, we tested our algorithm on two EEG datasets to check its reliability. These two datasets have been used widely during the past few years [18, 19]. To demonstrate the advantages of our method, it was rational to compare our method with existing wavelet-based algorithms by using these well-recognized datasets. All computational experiments are run on a server with 32-core AMD CPU (1400MHz) using Matlab 2013a (MathWorks, Natick, Massachusetts, U.S.A).

#### MIT dataset

The first dataset in this work was collected at the Children’s Hospital Boston, Massachusetts (MIT), consists of EEG recordings from pediatric subjects with intractable seizures. Subjects were monitored for up to several days following withdrawal of anti-seizure medication in order to characterize their seizures and assess their candidacy for surgical intervention [20]. Recordings were collected from 22 subjects (5 males, ages 3–22; and 17 females, ages 1.5–19). The International 10–20 system of EEG electrode positions and nomenclature was used for these recordings. More details about the dataset can be found from [18] and http://www.physionet.org/pn6/chbmit/.

EEG recordings in all channels from seizure start to end (ictal) were considered as “seizure”; EEG recordings out of the period of “seizure” were considered as “non-seizure”. Therefore, seizure detection could be further transformed into a signal classification problem: classifying seizure and non-seizure EEG signals from simultaneously recorded multi-channel EEG signals.

**Fig 1:**
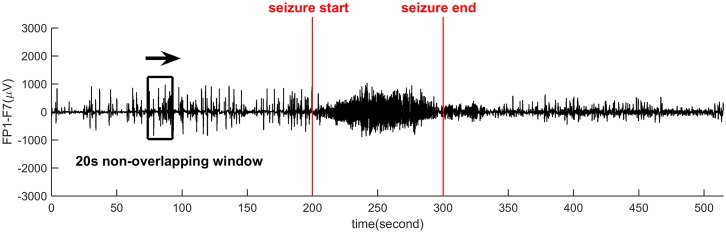
Seizure and non-seizure EEG segments from MIT dataset. A 20-second’ window slides across the long-time EEG. If the window goes into a period of seizure, this segment is marked as “seizure”, otherwise, “non-seizure”.

The EEG signals were sampled at 256Hz and digitally filtered by a 48th-order FIR high-pass filter (hamming window) with the cutoff frequency at 0.5Hz to remove low-frequency artifacts. The 256Hz sampling rate is large enough to cover general human EEG rhythms (bandwidths), including, *δ*(< 4*Hz*), *θ*(4 − 7*Hz*), *α*(8 − 15*Hz*), *β*(16 − 31*Hz*) and *γ*(> 31*Hz*). In this work, 13846 EEG segments were chosen from 18 cases (several subjects having much shorter seizure recordings than others were abandoned to keep the data balance), each segment lasts 20 seconds. Each subject provides the same number of seizure and non-seizure segments [21]. In total, 38.46h seizure EEG and 38.46h non-seizure EEG are used. The EEG segment selection is shown in Fig 1 which gives a 520 seconds EEG recording of a single channel. The 20 second non-overlapping window slides from left to right. When the slide windows falls into a seizure onset area (between the two read lines), the segment was selected as “seizure”. Otherwise, the segment was treated as “non-seizure”. Segments shorter than 20 second were discarded here.

#### UBonn dataset

The second dataset in this work was from University of Bonn (UBonn) [19]. The dataset had five sets denoted A∼E, each containing 100 single channel EEG segments of 23.6-sec duration with a sampling rate of 173.61 Hz. These segments were selected and cut out from continuous multichannel EEG recordings after visual inspection for artifacts. The scalp EEG signals were digitally filtered using a 48th-order FIR high-pass filter (hamming window) with the cutoff frequency at 0.5Hz.

For seizure detection, sets C, D were treated as “non-seizure” while set E was treated as “seizure”. In this study, we focused on seizure detection for patients. Sets C, D originated from EEG archive of presurgical diagnosis. Segments in set D were recorded from within the epileptogenic zone, and those in set C from the hippocampal formation of the opposite hemisphere of the brain. Sets C and D contained only activity measured during seizure-free intervals while set E only contains seizure activity.

### Framework

**Fig 2:**
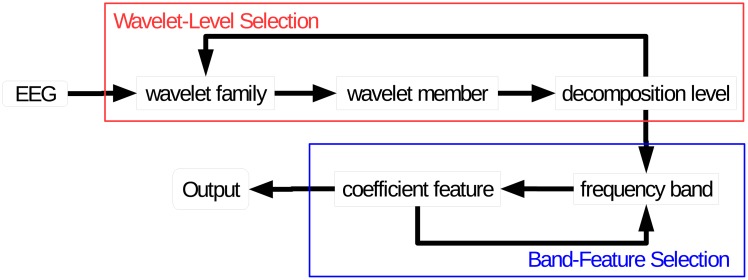
Framework of our method based on wavelet. The full algorithm can be divided into two parts. The Wavelet-Level Selection and the Band-Feature Selection. For each mother wavelet, one EEG segment is decomposed to the highest theoretical level for later feature extraction. For each wavelet family, only the mother wavelet and corresponding decomposition level, which produce the highest classification accuracy, is retained for Band-Feature Selection. In Band-Feature Selection, the features in certain bands leading to the highest accuracy are used to construct the final prediction model.

The framework of our seizure detection method based on wavelet is shown in Fig 2. Our algorithm was constructed by two main selection blocks, a Wavelet-Level Selection and a Band-Feature Selection. Long period of seizure and non-seizure EEGs were used, artifact contaminated EEGs were included. This high-performance algorithm was a completely automatic process. DWT was used to construct a feature vector for each EEG segment. A support vector machine (SVM) classifier [22] would learn to distinguish the feature vectors of seizure and non-seizure EEGs, automatically. Details inside were introduced in the following subsections.

### Discrete wavelet transform

DWT played a significant role in our algorithm. A wavelet is a quickly vanishing oscillating function localized both in frequency and time domains. In continuous wavelet analysis, the signal is decomposed into scaled and translated versions (*ψ*_*a*,*b*_(*t*)) of a single function *ψ*(*t*) called mother wavelet:

$$\psi_{a,b}\left(t\right)=\frac{1}{\sqrt{\left|a\right|}}\psi \left(\frac{t-b}{a}\right)$$ (1)

where *a* and *b* are the scale and translation parameters, respectively, with a,b∈R and *a* ≠ 0. The discrete wavelet transform (DWT) [23] was obtained by discretizing the parameters *a* and *b*. In its most common form, the DWT employs a dyadic sampling with parameters *a* and *b* based on powers of two: *a* = 2^*j*^ and *b* = *k*2^*j*^, with j,k∈Z. By substituting in Eq 1, we obtained the dyadic wavelets:

$$\psi_{j,k}\left(t\right)=2^{-j/2}\psi \left(2^{-j}t-k\right)$$ (2)

Of note, DWT could be written as

$$d_{j,k}=\int_{-\infty }^{+\infty }s\left(t\right)2^{-j/2}\psi^{*}\left(2^{-j}t-k\right)\;dt=\langle s\left(t\right),\psi_{j,k}\left(t\right)\rangle$$ (3)

where *d*_*j*,*k*_ are known as wavelet coefficients at level *j* and location *k* [24]. These coefficients were used to construct the feature vector of each EEG segment in seizure detection.

### Wavelet family & wavelet member

In wavelet-based digital signal processing (DSP), selecting a suitable mother wavelet [23] is always the first step. Various mother wavelets supply different DWT coefficients on the same EEG segment leading to different detection results. In this work, 7 commonly used wavelet families were tested, including, Biorthogonal (bior), Coiflets (coif), Daubechies (db), Reverse biorthogonal (rbio), Symlets (sym), Discrete Meyer (dmey), and Haar (Haar) [6]. Fifty-four family members (mother wavelets) totally contained in these families are shown in Table 1.

**Table 1:** Fifty-four Mother Wavelets.

Wavelet family	Mother wavelet
Biorthogonal (bior)	bior1.1, bior1.3, bior1.5, bior2.2, bior2.4, bior2.6, bior2.8, bior3.1, bior3.3, bior3.7, bior3.9, bior4.4, bior5.5, bior6.8
Coiflets (coif)	coif1, coif2, coif3, coif4, coif5
Daubechies (db)	db1, db2, db3, db4, db5, db6, db7, db8, db9, db10
Reverse biorthogonal (rbio)	rbio1.1, rbio1.3, rbio1.5, rbio2.2, rbio2.4, rbio2.6, rbio2.8, rbio3.1, rbio3.3, rbio3.7, rbio3.9, rbio4.4, rbio5.5, rbio6.8
Symlets (sym)	sym2, sym3, sym4, sym5, sym6, sym7, sym8
Discrete Meyer (dmey)	dmey
Haar (Haar)	haar

It is worth noting that in clinical practice, testing all wavelets is impractical and unnecessary. In addition, sometimes mother wavelets should be chosen according to the properties of patient EEG recordings. Heuristics for selecting mother wavelets are discussed in a later section.

### Decomposition level

Decomposition level is an important parameter of DWT. Each level in DWT corresponds to a specific frequency band. More levels of decomposition provide more detailed depictions of the signal, but may produce feature redundancy leading to accuracy reduction and computational cost increasing (sometimes exponentially, e.g., when using RBF kernel SVM [25] as the classifier).

The maximum level *L* of decomposition level is jointly determined by the signal and the mother wavelet to satisfy the condition:

$$L<\log_{2}\frac{N}{F-1}+1,$$ (4)

where *N* is the signal size and *F* is the filter size [26]. Each EEG segment has 5120 samples and 4097 samples, respectively, in MIT dataset and UBonn dataset. The corresponding maximum decomposition level of each wavelet in these two datasets are given in the following section.

### Frequency band

In DWT, each decomposition level corresponds to a certain frequency band. Supposing the raw EEG data would fall in frequency band (*a*, *b*), according to Mallat algorithm [27], at level *n*, the approximation frequency band would be:

$$\left(a,a+\frac{b-a}{2^{n}}\right)$$ (5)

the detail frequency band is

$$\left(a+\frac{b-a}{2^{n}},a+\frac{b-a}{2^{n-1}}\right)$$ (6)

**Fig 3:**
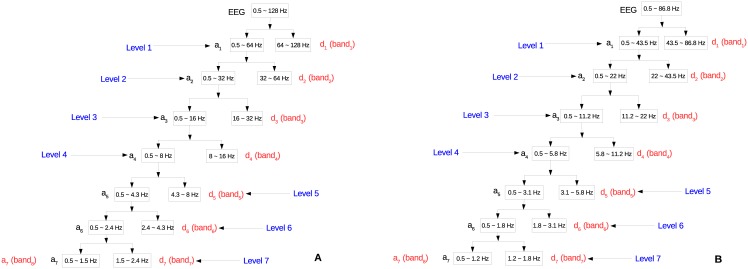
Examples of 7-level decomposition and corresponding frequency bands. (A) On MIT dataset. (B) On UBonn dataset. The EEG signals are decomposed into several frequency bands. *d*_*i*_ is the detail band while *a*_*i*_ (*i* = 1, 2, … 7) is the approximation band. All detail bands and the last approximation band might be used for feature extraction.

Fig 3 illustrates the frequency bands covered by each level of decomposition on MIT and UBonn datasets, given the frequency range (0.5, 128)*Hz* for MIT and (0.5, 86.8)*Hz* for UBonn. In this figure, the detail band and approximation band on the *i*^th^ decomposition level are denoted as *d*_*i*_ and *a*_*i*_ (*i* = 1, 2, … 7), respectively. As to be discussed later, wavelet coefficients of several bands, as shown with red annotations in the figure, construct the feature vector for each EEG segment.

In clinical practice, EEG is typically described in terms of rhythmic activity, which means in DWT-based EEG analysis; a specific frequency band corresponds to a certain EEG rhythm. “Seizure” and “non-seizure” EEG segments might have significant difference in certain frequency bands. EEG segments could be classified accurately by features from these bands. However, some frequency bands should be abandoned since features from these bands caused redundancy and accuracy reduction. This issue is considered in later Band-Feature Selection to improve accuracy and reduce feature vector redundancy.

### Coefficient feature

Choosing suitable features that can best represent the characteristics of the EEG signals is important for EEG classification [11]. DWT coefficient features from several frequency bands construct the feature vector of one EEG signal segment.

In this study, the DWT coefficients of an EEG segment in each band were calculated according to Eq 3. Seven commonly used wavelet features in wavelet-based EEG signal processing and two statistical features constructed the feature vector of each EEG segment. These features are indicated in Table 2.

**Table 2:** Features in Each Band.

Feature Name	Description
Max	the maximum coefficient
Min	the minimum coefficient
Mean	the mean of coefficients
STD	the standard deviation of coefficients
skewness	the skewness of coefficients
kurtosis	the kurtosis of coefficients
Energy	the squared sum of all coefficients
nSTD	normalized standard deviation, $\frac{STD}{Max-Min}$
nEnergy	normalized energy (ratio between energy and the size of the band)

### Classification

Seizure detection is formulated into a binary classification problem on two kinds of EEG segments, “seizure” and “non-seizure”. SVM with RBF kernel was used as the classifier. Here we briefly go over the concepts of binary classification and SVM. SVM is a supervised learning algorithm that can be used for binary classification. A SVM constructs an optimal hyperplane as a decision surface such that the margin of separation between the two classes in the data is maximized. Support vectors refer to a small subset of the training observations that are used as support for the optimal location of the decision surface. Only the support vectors chosen from the training data are required to construct the decision surface. Details of SVM and binary classification could be found from previous work [22].

To assess the performance of our approach, especially its ability to overcome individual difference, we used leave-one-subject-out cross-validation on MIT dataset. Each time, only one subject’s data was used as the test set while all others’ data as the training set. Mixing one subject’s data in both training and test sets might give the algorithm prior knowledge and cause false high accuracy. Hence, leave-one-subject-out cross-validation was a fair evaluation scheme to truly reveal the robustness of the classifier on overcoming the individual difference. Since UBonn dataset did not separate the data from different patients, 10-fold cross validation was used instead of leave-one-subject-out.

In this paper, “seizure” EEG segments were considered as “positive” while “non-seizure” segments were considered as “negative”. Therefore, the classifier had 4 possible outcomes [6]:

1. True positive (*TP*);
2. False positive (*FP*);
3. True negative (*TN*);
4. False negative (*FN*).

As listed in Table 3, five values in the confusion matrix are employed to evaluate the algorithm performance, including, Accuracy, Sensitivity, Specificity, Positive Predictive Value (PPV), and Negative Predictive Value (NPV).

**Table 3:** Confusion Matrix.

		Predicted condition		
	Total population	Predicted condition positive	Predicted condition negative	
True condition	Condition positive	True positive (TP)	False negative (FN)	$\text{True positive rate}(\text{TPR}),\text{Sensitivity}=\frac{\sum Ture\;positive}{\sum Condition\;positive}$
	Condition negative	False positive (FP)	True negative (TN)	$\text{True negative rate}(\text{TNR}),\text{Specificity}=\frac{\sum Ture\;negative}{\sum Condition\;negative}$
	$\text{Accuracy}=\frac{TP+TN}{TP+FP+TN+FN}$	$\text{Positive predictive value}(\text{PPV})=\frac{\sum Ture\;positive}{\sum Test\;outcome\;positive}$	$\text{Negative predictive value}(\text{NPV})=\frac{\sum Ture\;negative}{\sum Test\;outcome\;negative}$	

### Wavelet-level selection

Mother wavelet and decomposition level are two factors that affect the performance of DWT in digital signal processing. By appropriately selecting the mother wavelet and decomposition level, DWT could accurately interpret the characteristics of the original EEG segment. Considering this, Wavelet-Level Selection was used for exploring the performance of each wavelet with all possible decomposition levels.

Wavelet-Level Selection was done as follows. Supposing a mother wavelet whose maximum decomposition level was *j*, DWT could divide the EEG segment into several bands with the number from 2 (1 detail bands and 1 approximation band) to *j* + 1 (*j* detail bands and 1 approximation band). For each mother wavelet and corresponding decomposition level, features across all the frequency bands constructed the feature vector for each EEG segment. In each wavelet family, the mother wavelet and related decomposition level leading to the highest seizure detection accuracy would be selected for later analysis. For each combination of wavelet and decomposition level, a cross-validation was performed by SVM.

### Band-feature selection

EEG is typically described in terms of rhythmic activity making different frequency bands in DWT corresponding to various EEG rhythms. In a certain EEG dataset, seizure and non-seizure EEG segments might provide a significant difference of rhythmic activity in the specific frequency band(s). Similar to frequency bands, in a certain EEG dataset, some features might help to distinguish seizure and non-seizure EEG segments while other features only generated data redundancy. Considering this, we framed a Band-Feature Selection, which explored the band(s) and the feature(s) that most precisely classified seizure and non-seizure EEG signals with low computational cost.

The Band-Feature Selection was done as follows. Given a mother wavelet whose best decomposition level of an EEG segment was *j*, DWT would divide the frequency range from 0Hz to half of the sampling rate into *j* + 1 bands (*j* detail bands and 1 approximation band). Hence, there were $\sum_{i=1}^{j+1}\left(\begin{array}{c} j+1\\ i \end{array}\right)$ combinations of bands. If each band had *m* features, there were $\sum_{n=1}^{m}(\begin{array}{c} m\\ n \end{array})$ combinations of features. As a result, for this mother wavelet and corresponding decomposition level, we had a total of $\sum_{i=1}^{j+1}\left(\begin{array}{c} j+1\\ i \end{array}\right)\cdot \sum_{n=1}^{m}(\begin{array}{c} m\\ n \end{array})$ combinations of bands and features. For each combination of band(s) and feature(s), a cross-validation was performed by SVM.

## Results

Results of Wavelet-Level Selection showed the effect of mother wavelet and decomposition level on DWT-based seizure detection. The results of Band-Feature Selection enabled us to improve seizure detection accuracy and remove feature redundancy.

### Wavelet-level selection

Both datasets, using suitable wavelet and decomposition level, provide promising seizure detection accuracy (results are summarized in Table 4). On MIT dataset, decomposition level affects the accuracy substantially regardless of the mother wavelets. On UBonn dataset, all wavelets could achieve high accuracy (above 95%) at low decomposition level (less than 2). The results on these two datasets were discussed separately.

**Table 4:** Wavelet member, maximum decomposition level, best decomposition level and corresponding accuracy.

	Wavelet	Max level	Best (Accuracy / Level)	Wavelet	Max level	Best (Accuracy / Level)	Wavelet	Max level	Best (Accuracy / Level)
MIT	bior1.1	12	88.09% / 8	coif4	7	88.71% / 7	rbio2.8	8	88.85% / 7
	bior1.3	10	88.12% / 10	coif5	7	88.77% / 7	rbio3.1	10	88.05% / 7
	bior1.5	9	88.05% / 9	db1	12	88.09% / 8	rbio3.3	9	88.91% / 7
	bior2.2	10	88.87% / 8	db2	10	88.60% / 7	rbio3.5	8	88.99% / 8
	bior2.4	9	88.73% / 7	db3	10	88.52% / 10	rbio3.7	8	88.79% / 8
	bior2.6	8	88.71% / 7	db4	9	88.65% / 8	rbio3.9	8	88.46% / 8
	bior2.8	8	88.86% / 7	db5	9	88.96% / 7	rbio4.4	9	88.55% / 6
	bior3.1	10	87.33% / 5	db6	8	88.75% / 6	rbio5.5	8	88.52% / 7
	bior3.3	9	88.42% / 7	db7	8	88.33% / 7	rbio6.8	8	88.97% / 7
	bior3.5	8	88.40% / 7	db8	8	89.00% / 7	sym2	10	88.60% / 7
	bior3.7	8	88.69% / 8	db9	8	88.81% / 7	sym3	10	88.52% / 10
	bior3.9	8	88.60% / 7	db10	8	89.03% / 7	sym4	9	88.70% / 8
	bior4.4	9	88.81% / 7	rbio1.1	12	88.09% / 8	sym5	9	88.91% / 8
	bior5.5	8	88.75% / 7	rbio1.3	10	88.52% / 7	sym6	8	88.88% / 7
	bior6.8	8	89.00% / 7	rbio1.5	9	88.44% / 8	sym7	8	88.87% / 7
	coif1	10	88.59% / 8	rbio2.2	10	88.58% / 6	sym8	8	88.90% / 7
	coif2	8	88.88% / 7	rbio2.4	9	88.69% / 7	dmey	5	87.32% / 5
	coif3	8	89.03% / 7	rbio2.6	8	88.57% / 7	haar	12	88.09% / 8
UBonn	bior1.1	12	96.67% / 1	coif4	7	97.00% / 2	rbio2.8	7	95.33% / 1
	bior1.3	9	97.00% / 1	coif5	7	96.67% / 2	rbio3.1	10	97.67% / 1
	bior1.5	8	96.67% / 1	db1	12	97.67% / 1	rbio3.3	9	95.00% / 1
	bior2.2	9	96.00% / 1	db2	10	95.67% / 1	rbio3.5	8	95.67% / 2
	bior2.4	8	96.00% / 1	db3	9	95.00% / 1	rbio3.7	8	96.00% / 2
	bior2.6	8	95.67% / 1	db4	9	96.33% / 2	rbio3.9	7	96.00% / 2
	bior2.8	7	96.00% / 1	db5	8	96.33% / 2	rbio4.4	8	95.00% / 1
	bior3.1	10	96.67% / 2	db6	8	95.33% / 1	rbio5.5	8	95.33% / 1
	bior3.3	9	96.33% / 2	db7	8	97.00% / 2	rbio6.8	7	95.67% / 2
	bior3.5	8	97.00% / 2	db8	8	95.33% / 1	sym2	11	95.67% / 1
	bior3.7	8	96.33% / 2	db9	7	95.00% / 1	sym3	11	95.00% / 1
	bior3.9	7	95.00% / 1	db10	7	95.00% / 1	sym4	10	95.00% / 1
	bior4.4	8	95.67% / 2	rbio1.1	12	96.67% / 1	sym5	10	97.00% / 2
	bior5.5	8	96.00% / 2	rbio1.3	9	96.00% / 1	sym6	9	95.00% / 1
	bior6.8	7	96.00% / 2	rbio1.5	8	96.67% / 2	sym7	9	95.00% / 1
	coif1	9	96.00% / 1	rbio2.2	9	96.00% / 1	sym8	9	96.67% / 2
	coif2	8	96.33% / 2	rbio2.4	8	95.00% / 1	dmey	5	96.33% / 2
	coif3	7	95.00% / 1	rbio2.6	8	95.00% / 1	haar	12	96.67% / 1

On MIT dataset, the best member of each family and its optimal decomposition level (i.e., the level that yielded to the highest accuracy) were used for Band-Feature Selection. On UBonn dataset, since high accuracy could be achieved at low decomposition level, the wavelet and the lowest decomposition level that achieved accuracy above 95% were of interest in Band-Feature Selection. In a certain wavelet family, if several wavelets achieved accuracies above 95% at the same decomposition level, the one having the smallest vanishing moment were selected.

#### MIT dataset

Our method delivered promising performance for seizure detection after Wavelet-Level Selection. Table 4 shows consistent accuracy above 87% across all 54 wavelets. Since we used leave-one-subject-out cross validation, our approach could overcome individual differences and build robust models.

**Fig 4:**
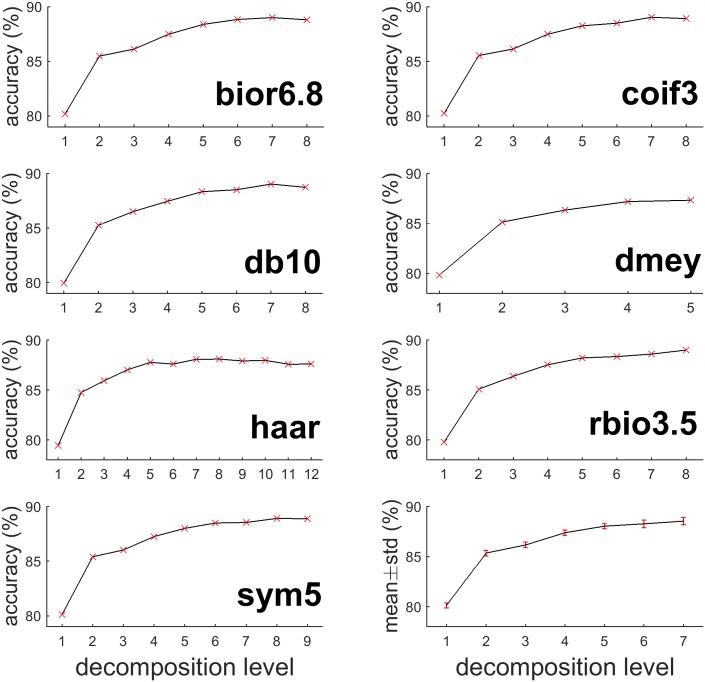
Relationship between seizure detection accuracy and decomposition level (MIT). X-axis is decomposition level while Y-axis is classification accuracy. The first 7 subplots indicate the mother wavelet, which produce the highest accuracy in corresponding wavelet family. The last subplot is the average accuracy of the 54 mother wavelets with an error bar of one standard deviation.

Results in this part showed a consistent pattern across all mother wavelets that more levels of decomposition brought little accuracy improvement beyond a certain level. Fig 4 shows how accuracy changes as decomposition level increases from 1 to maximum levels on 7 mother wavelets that performed the best in their families. As decomposition level increased, seizure detection accuracy was significantly improved. However, after the level increased to about half of the maximum level, the accuracy improvement becomes very limited. In many cases, the accuracy even dropped (e.g., bior6.8, db10 and coif3 reached the accuracy peaks at level 7). Pursuing more levels of decomposition, which increases computational cost, would not necessarily improve performance.

Table 4 shows that giving suitable decomposition level for each mother wavelet can yield similar accuracy for all wavelets. Most wavelets reached accuracy above 87% when decomposition reached to level 7 or above. The highest classification accuracy was 89.03% when using coif3 decomposed to level 7. The bottom right subplot in Fig 4 shows the average detection accuracy with one standard deviation of different wavelets at level 1 to 7 (dmey is excluded because its maximum decomposition level is only 5). The average accuracy ascended with decomposition level, with the extreme value attained at level 7 (88.54%). The standard deviation retained low value (less than 0.3%), indicating that seizure detection accuracy was more sensitive to decomposition level, but not mother wavelet.

#### UBonn dataset

Compared with the results in MIT dataset, EEG segments in UBonn could be accurately classified at very low decomposition level. All wavelets provided seizure detection accuracy near to 100%. The lowest level that attains the accuracy above 95% are shown in Table 4. In each wavelet family, the wavelet member having the smallest vanishing moment achieved the accuracy above 95% was selected for analysis in a later section.

**Fig 5:**
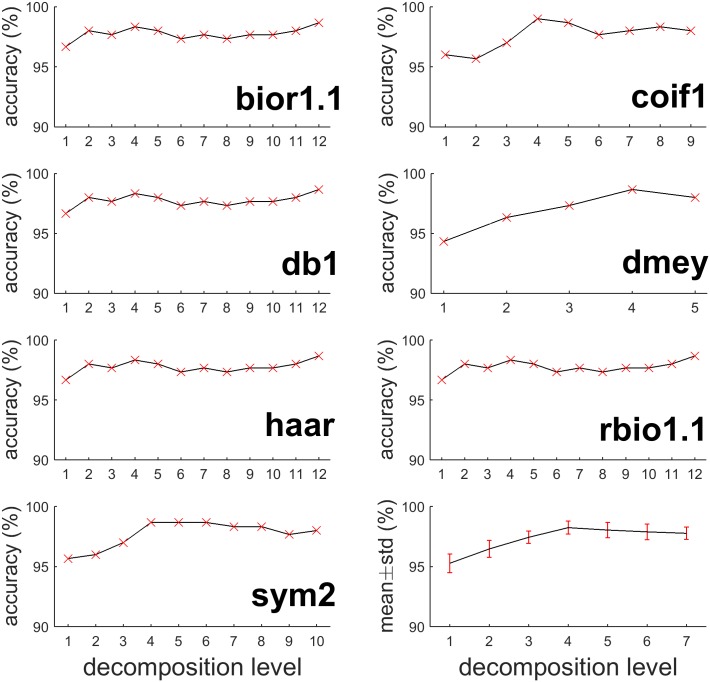
Relationship between seizure detection accuracy and decomposition level (UBonn). X-axis is decomposition level while Y-axis is classification accuracy. The first 7 subplots indicate the mother wavelet, which produce the highest accuracy in corresponding wavelet family. The last subplot is the average accuracy of the 54 mother wavelets with an error bar of one standard deviation.

We used 95% as an accuracy threshold for later analysis, it is still worth to investigate the relationship between accuracy and decomposition level on this dataset. The bottom right subplot in Fig 5 shows the average detection accuracy with one standard deviation of different wavelets at level 1 to 7 (dmey was excluded because its maximum decomposition level was only 5). The average accuracy ascended as decomposition level increased and arrived the extreme value at level 4 (98.24%). The standard deviation in UBonn dataset retained low value (less than 0.78%), indicated the same conclusion as in MIT dataset that seizure detection accuracy was also more sensitive to decomposition level, but not mother wavelet.

### Band-feature selection

After selecting wavelet member and decomposition level, Band-Feature Selection extracted the frequency bands and features leading to the optimal seizure detection accuracy with low computational cost.

#### MIT dataset

**Fig 6:**
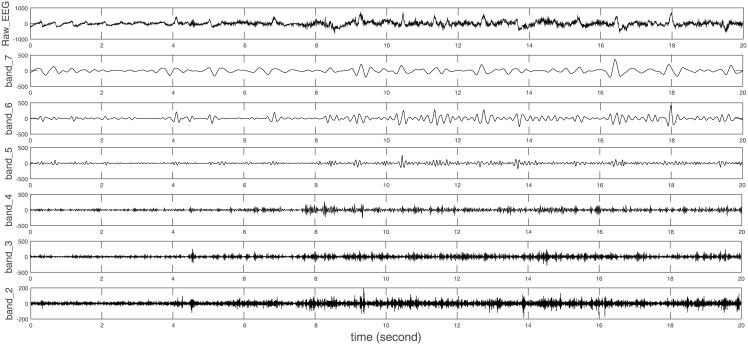
Single-level reconstruction of DWT for one seizure segment on MIT dataset. Among all wavelets, coif3 [28] achieves the highest accuracy of 92.30% using 7 features from 6 bands. An example of the single-level reconstruction of the 6 bands are shown here. The IDs of the bands are given in Fig 3.

The Band-Feature Selection was done on the 7 wavelets that performed the best in experiments above in 7 respective families. The best bands, features, and confusion matrix values are given in Table 5. Among all wavelets, coif3 [28] achieves the highest accuracy of 92.30% using 7 features from 6 bands. The details of its single-level reconstruction details are shown in Fig 6.

**Table 5:** Result of Band-Feature Selection.

	Wavelet(best in family)	Accuracy	Sensitivity	Specificity	PPV	NPV	Features	Bands	Dimensionality Reduction
MIT	bior6.8	89.01%	88.39%	89.62%	89.49%	88.53%	Max, Min, Mean, STD, skewness, Energy, nSTD, nEnergy	2–7	33.33%
	coif3	92.30%	91.71%	92.89%	92.80%	91.80%	Max, Min, Mean, STD, skewness, Energy, nSTD	2–7	41.67%
	db10	89.08%	88.67%	89.49%	89.40%	88.76%	Max, Min, Mean, STD, skewness, Energy, nSTD	2–7	41.67%
	dmey	87.93%	88.40%	87.46%	87.57%	88.29%	Max, Min, Mean, STD, skewness, Energy, nSTD, nEnergy	2–5	40.74%
	haar	88.21%	88.08%	88.33%	88.30%	88.11%	Min, STD, skewness	2–9	70.37%
	rbio3.5	89.48%	88.64%	90.31%	90.15%	88.83%	Max, Min, STD, skewness	2–9	60.49%
	sym5	89.05%	88.98%	89.12%	89.10%	88.99%	Max, Min, Mean, STD, skewness, Energy, nSTD, nEnergy	2–9	20.99%
UBonn	bior1.1	98.67%	98.00%	99.00%	98.00%	99.00%	Max, STD, kurtosis, Energy	2	77.78%
	coif1	96.00%	96.00%	96.00%	92.31%	97.96%	Min, skewness, kurtosis, Energy	2	77.78%
	db1	98.67%	98.00%	99.00%	98.00%	99.00%	Max, STD, kurtosis, Energy	2	77.78%
	dmey	98.67%	99.00%	98.50%	97.06%	99.49%	STD, skewness, Energy, nSTD, nEnergy	2, 3	62.96%
	haar	98.67%	98.00%	99.00%	98.00%	99.00%	Max, STD, kurtosis, Energy	2	77.78%
	rbio1.1	98.67%	98.00%	99.00%	98.00%	99.00%	Max, STD, kurtosis, Energy	2	77.78%
	sym2	99.33%	97.00%	100.00%	100.00%	98.52%	Max, STD, kurtosis, Energy, nSTD	2	72.22%

PPV: Positive predictive value; NPV: Negative predictive value.

The IDs of bands follow the Eqs 5 and 6, reference is given in Fig 3.

Considering the computational cost, haar was also a good choice. haar achieved 88.21% accuracy when using only 3 features from 8 bands that required much shorter feature vector than other wavelets. Table 5 show the features and bands that yield the highest accuracy for each wavelet (see columns “Features” and “Bands”).

#### UBonn dataset

The lower half of Table 5 lists the choice of bands, features and mother wavelets that yielded the highest accuracy in each wavelet family after Band-Feature Selection on UBonn dataset. The highest accuracy after Band-Feature Selection was 99.33% with 5 features in 1 frequency band under sym2.

In Table 5, column “Features” shows features that yield the highest accuracy for each wavelet. Since all the wavelets provide high accuracy at low decomposition level (≤ 2), the band selection results in UBonn is definitely 1 or 1 & 2.

### Comparison with other works

**Fig 7:**
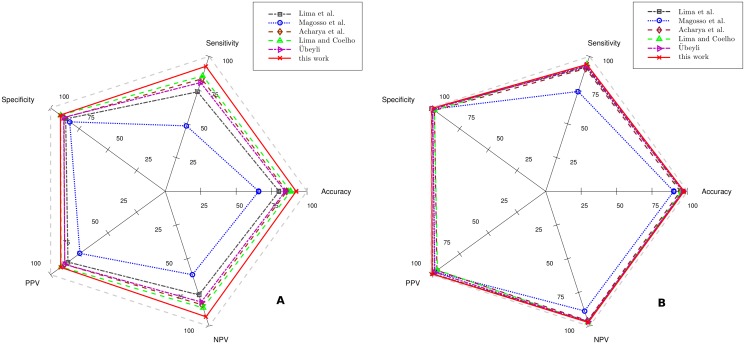
Radar chart of six methods’ performance in seizure detection. (A) On MIT dataset. (B) On UBonn dataset. The output of each method is represented by a pentagon in specific color. The outermost grey line means the 100% accuracy of the five values in confusion matrix. A better method should achieve a larger pentagon area.

Our method provided promising performance in seizure detection. We further compared our algorithm with existing methods. Fig 7, a radar chart, shows the comparison of our method and five commonly used seizure detection methods based on DWT.

Confusion matrix values of MIT and UBonn datasets are given in Table 6. Although each method could use various mother wavelets with many parameters settings, only the configuration that yielded to the highest accuracy for each method is shown in Fig 7.

**Table 6:** Comparison with other works.

	Method	Accuracy	Sensitivity	Specificity	PPV	NPV
MIT	Lima et al. [29]	80.30%	73.74%	86.85%	84.88%	76.81%
	Magosso et al. [24]	65.92%	48.50%	83.34%	74.44%	61.81%
	Acharya et al. [30]	85.00%	83.31%	88.29%	87.69%	84.12%
	Lima and Coelho [31]	88.45%	85.59%	91.32%	90.81%	86.38%
	Übeyli [32]	84.60%	80.62%	88.58%	87.61%	82.06%
	this work (coif3)	92.30%	91.71%	92.89%	92.80%	91.80%
UBonn	Lima et al. [29]	97.00%	92.00%	99.50%	98.75%	96.30%
	Magosso et al. [24]	90.33%	74.00%	98.50%	96.64%	88.68%
	Acharya et al. [30]	95.00%	91.00%	97.00%	94.85%	95.89%
	Lima and Coelho [31]	96.00%	94.00%	97.00%	94.42%	97.11%
	Übeyli [32]	96.67%	93.00%	98.50%	96.98%	96.69%
	this work (sym2)	99.33%	97.00%	100.00%	100.00%	98.52%

PPV: Positive predictive value; NPV: Negative predictive value.

In the radar chart, the better method gives a larger area of the pentagon whose 5 vertexes correspond to the 5 values in confusion matrix, including, accuracy, sensitivity, specificity, PPV, and NPV. Our method was noticeably superior to other methods since it had the largest areas on both MIT and UBonn datasets. Using our algorithm, all confusion matrix values based on our method were higher than 90.00%. The maximum difference of confusion matrix values between the two datasets was only 7.20% on PPV. In contrast, though existing methods performed well on UBonn dataset, their performance on MIT dataset was poor. Take Lima et al. [29] for example. Though its confusion matrix values were all above 90.00% on UBonn, these values drop sharply (at least 12.65% on specificity) when processing EEG segments in MIT dataset. Results showed that our method was more accurate and transferable on seizure detection.

## Discussion

### Wavelet-level selection

Empirical results reveal the effects of four factors in DWT-based seizure detection. For some applications, specific properties of wavelet, i.e., symmetry, interpolating scaling functions, or dyadic rational filter coefficients [28], may cause nuisance. However, in other cases, including our work here, these properties of wavelet do not matter at all [33]. On dataset having complex EEG signals (contain hidden information distribution in several frequency bands), like MIT dataset, decomposition level influences accuracy substantially regardless of the mother wavelet. Choosing a suitable decomposition level, all mother wavelets provide similar seizure detection accuracies. On dataset having easy-classified EEG segments, like UBonn dataset, the seizure detection accuracy is sensitive to neither mother wavelet nor decomposition level.

### Band-feature selection

Band-Feature Selection can increase the seizure detection accuracy and reduce feature vector dimension greatly on both datasets in this work. It achieved an accuracy increase of 3.27% (using coif3) and 3.63% (using sym2), respectively, on MIT dataset and UBonn dataset. In addition, Band-Feature Selection removed redundant features to save computational cost. On MIT dataset, take haar for example, the feature vector dimension was reduced from 81 = 9 × 9 (8 detail bands, 1 approximation band, 9 features in each band) to 24 = 8 × 3 (8 detail bands, 3 features in each band). On UBonn, take sym2 for example, the feature vector dimension was reduced from 18 = 2 × 9 (1 detail band, 1 approximation band, 9 features in each band) to 5 = 1 × 5 (1 detail band, 5 features). The results indicate that our new algorithm can efficiently minimize redundant features in useless frequency bands.

#### MIT dataset

**Fig 8:**
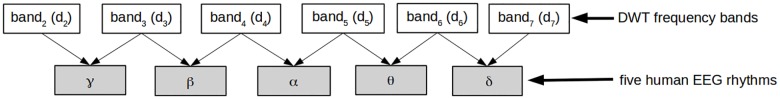
Mapping between bands in DWT and human EEG rhythms on MIT dataset. Each DWT frequency band relates to one or two human EEG rhythm. The frequency bands are calculated according to Eqs 5 and 6.

In MIT dataset, features *Max*, *Min*, *Mean*, *STD*, *skewness*, share the same feature selection result on more than 6 wavelets. This means seizure and non-seizure EEGs have the most significant differences on these features. *Kurtosis* is not chosen in any case which means the wavelet features of seizure and non-seizure EEG have no significant peak or tail difference. Bands across all conventional human EEG rhythms yield to best performance. A pattern can be found from the correspondence between those bands and EEG rhythms which are *δ*(< 4*Hz*), *θ*(4 − 7*Hz*), *α*(8 − 15*Hz*), *β*(16 − 31*Hz*) and *γ*(> 31*Hz*). The best accuracy can be achieved when there are always two DWT bands covering one EEG rhythm. For example, DWT bands 2 to 7 are best bands for wavelet coif3. The correspondence between the DWT bands of coif3 and EEG rhythms is illustrated in Fig 8. Such a pattern still holds for other wavelets. Our explanation of the results is that seizure and non-seizure EEG differs the most in conventional human EEG rhythms.

In this part, most deep DWT bands (e.g., bands 10 to 13 for haar) are abandoned. This phenomenon could be explained by the property of wavelet transform. Deep DWT bands correspond to low frequency EEG such as *δ* rhythm. Taking rbio1.1 for example, according to Mallat algorithm [27], a 20-second EEG signal which spans over the band (0.5, 128)Hz has the maximum number of bands at 13, including 12 detail bands and 1 approximation band. All bands after level 6 fall into the *δ* band, causing an over-representation of the *δ* band which could “confuse” the classifier. Hence, deep bands are abandoned by Band-Feature Selection in MIT dataset in most cases.

Results in Table 5 also show that for many wavelets, band 1 (64–128Hz) does not contribute to accuracy. Because the upper bound of *γ* rhythm is usually considered between 80Hz and 100Hz, we can hypothesize that seizure and non-seizure EEGs exhibit their major difference in conventional EEG rhythms. Recently, some studies have proven that EEGs in high-frequency oscillation (HFO) [34] will be a new area for epilepsy-related research. High frequency recordings could detect spikes or fast waves related to seizure, which might be missed in traditional human EEG bandwidths. It will be interesting to study whether it is still the case in high-frequency oscillation (HFO) in the future.

#### UBonn dataset

Experimental results indicate that the Band-Feature Selection produce different outputs in MIT and UBonn datasets. This difference is probably caused by EEG data structure. The useful features may lead to high seizure detection accuracy vary among different patients and EEG recording devices. Some features on MIT like *Min*, *Mean* and *skewness* that can describe the raw EEG of patients precisely might be useless in UBonnm. Vice versa, the feature *kurtosis* and *Energy* are not chosen in any case in MIT while they are picked up in most cases in UBonn. This indicates that EEG samples in MIT have no significant difference at *kurtosis* and *Energy*, while seizure and non-seizure EEG segments in UBonn are easy to be classified by these two features. Different EEG datasets also generate various frequency characteristics. Selected frequency bands in MIT cross all the conventional human rhythms while results in UBonn contain only 1 or 2 bands. This indicates that EEG segments in MIT are much more complex than UBonn’s. Using features from only 1 or 2 bands cannot classify seizure and non-seizure EEG segments accurately on MIT dataset.

### Suggestion in seizure detection using DWT

**Fig 9:**
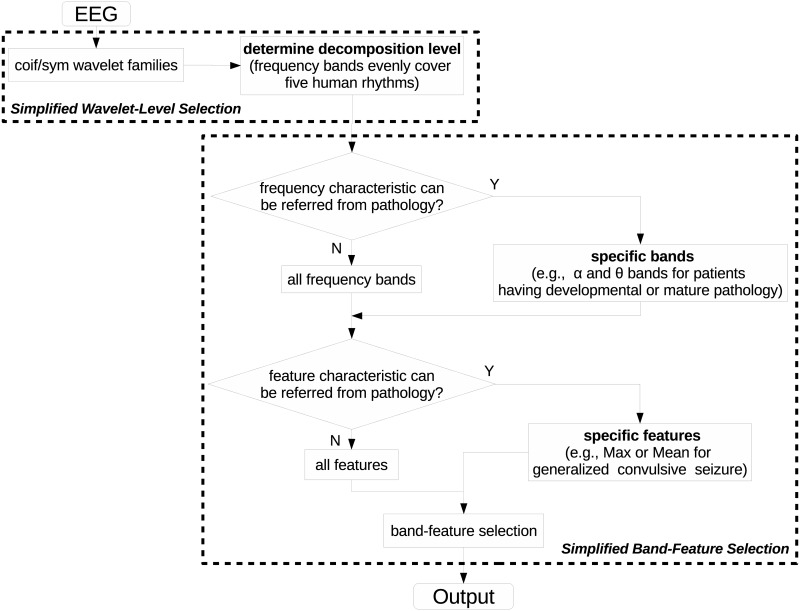
Suggested process in seizure detection using DWT. We suggest using coif and sym as two options since these two wavelet families have much better performance than the others. A decomposition level making the DWT frequency bands to cover the five human rhythms is high enough for clinical practice. Furthermore, in Band-Feature Selection, if EEGs from patient show specific frequency characteristic, the selection of band could be done among certain frequency bands. Similarly, specific features of patient’s EEGs can also be used to save computational cost.

Although our approach is fully automatic, running the process in Fig 2 is very time-consuming, especially when using various wavelet families and long-time continuous EEG segments. Therefore, in Fig 9, we suggest some heuristics of using DWT for seizure detection to simplify the process in Fig 2.

Despite the diversity of wavelet families and wavelet members, in seizure detection, it is futile to test many wavelet families (or members) since most wavelets provide similar accuracies. We should pay more attention to decomposition level as it plays a more important role than wavelet. Depend on previous discussion; the Wavelet-Level Selection could be simplified as in the upper dash block in Fig 9. Coif and sym are generally preferred for their good performance demonstrated in our experiment. For guaranteeing a high reliable algorithm, the decomposition level should at least evenly cover all conventional human EEG rhythms.

The Band-Feature Selection block could be simplified as in the lower dash block in Fig 9. After determining the decomposition level, frequency bands should be selected in two ways. The first one depends on patient pathology. For example, if patients have developmental or mature pathology [35], frequency bands in *α* and *θ* rhythms are more significant than other bands as alpha-theta sinusoidal and spike waves are easy to catch during seizure onset under this pathology. Extracting features from two frequency bands will definitely use much less time than a similar procedure on five bands. The second one should be used when patient pathology is unclear or complexly hidden inside several frequency bands. Under this circumstance, we suggest to select all the frequency bands after Wavelet-Level Selection. Similar to frequency bands, patient pathology may give reference in selecting DWT coefficient features. For example, if the patient has generalized convulsive seizure [36], using feature *Max* or *Mean* could efficiently distinguish “seizure” from “non-seizure” EEG segments of this kind of seizure is usually accompanied with high amplitude discharges in brain. Otherwise, testing more features may produce better result. Band-Feature Selection here is the same as in Fig 2 if both the decision blocks give negative outputs. Otherwise, the computational cost in this procedure could be reduced efficiently.

## Conclusion

Because of its adaptive resolution property for different frequencies, wavelet transform is gaining its ground in epilepsy-related EEG classification. However, how to efficiently use wavelet in EEG-based seizure detection is still an unanswered question. In this study, we explored the optimal combination of four factors, mother wavelet, decomposition level, frequency band, and feature. Efficiently setting these factors will lead to high seizure detection accuracy with low computational cost. Experimental results show that the accuracy is very sensitive to decomposition level regardless of the mother wavelet when classifying some complex EEG signals. Otherwise, the accuracy is sensitive to neither of them. Due to the structure difference between EEG datasets, various features and frequency bands have different significances in seizure detection. The Band-Feature Selection can abandon redundant features and frequency bands to improve detection accuracy and computation efficiency greatly.
